# A Rare Case of Angioleiomyoma of the Hand

**DOI:** 10.7759/cureus.7530

**Published:** 2020-04-03

**Authors:** Hiffsa Taj, Isin Comba, Jonathan Vasquez, Vania Zayat

**Affiliations:** 1 Internal Medicine, University of Central Florida College of Medicine, Orlando, USA; 2 Pathology, Orlando Veterans Affairs Medical Center, Orlando, USA; 3 Pathology, University of Central Florida College of Medicine, Orlando, USA

**Keywords:** angioleiomyoma, painless mass of hand, hand, tumor, histopathology, subcutaneous nodule

## Abstract

Angioleiomyoma is a benign tumor of the vascular system that is often not considered in the differential diagnosis of a painless subcutaneous nodule of the body. In this report, we present a rare case of angioleiomyoma of the phalanx in the left hand in a middle aged man. He underwent surgical excision of the mass with no complications. Given the nonspecific and indolent presentation, our case had a broad differential diagnosis including ganglion cyst and histopathological examination was warranted for definite diagnosis. At 9-month follow-up, the patient was asymptomatic with no signs of recurrence.

## Introduction

Angioleiomyoma, also known as “vascular leiomyoma,” is a benign tumor derived from the muscular tissue in tunica media layer of the vascular system. Although it can arise anywhere in the body, finger involvement is a very rare entity [1,2]. It typically presents as a slowly growing subcutaneous mass with pain being the most common presenting symptom [1,3]. The typical management involves surgical excision of the mass with close follow-up. In this report, we present a rare case of angioleiomyoma of the phalanx in the left hand. Given the nonspecific and indolent presentation, our case had a broad differential diagnosis and histopathological examination was warranted for definite diagnosis.

## Case presentation

**Figure 1:**
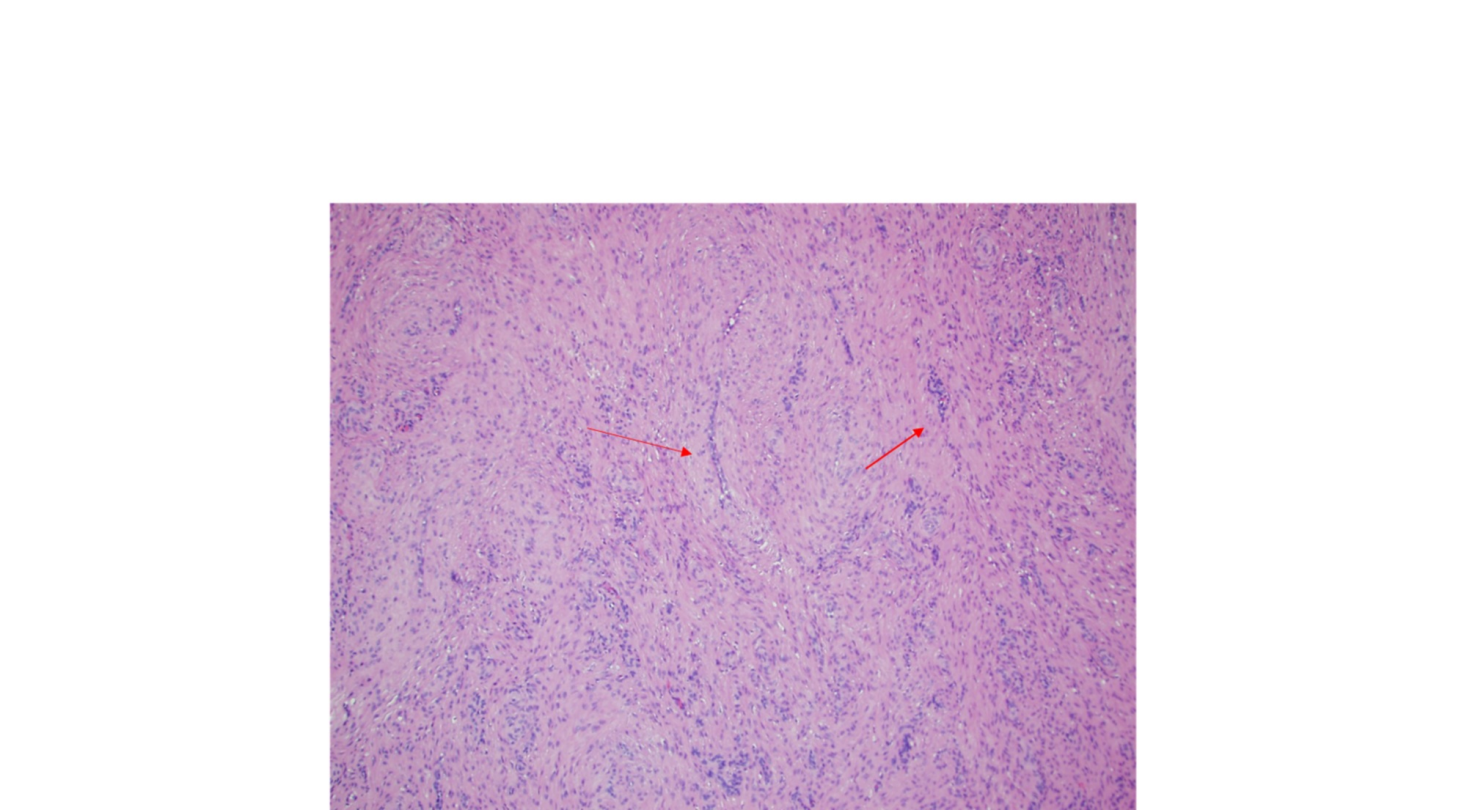
Microscopic exam revealed closely packed fascicles of spindle cell with myoid features surrounding compressed vascular lumina (10x)

**Figure 2:**
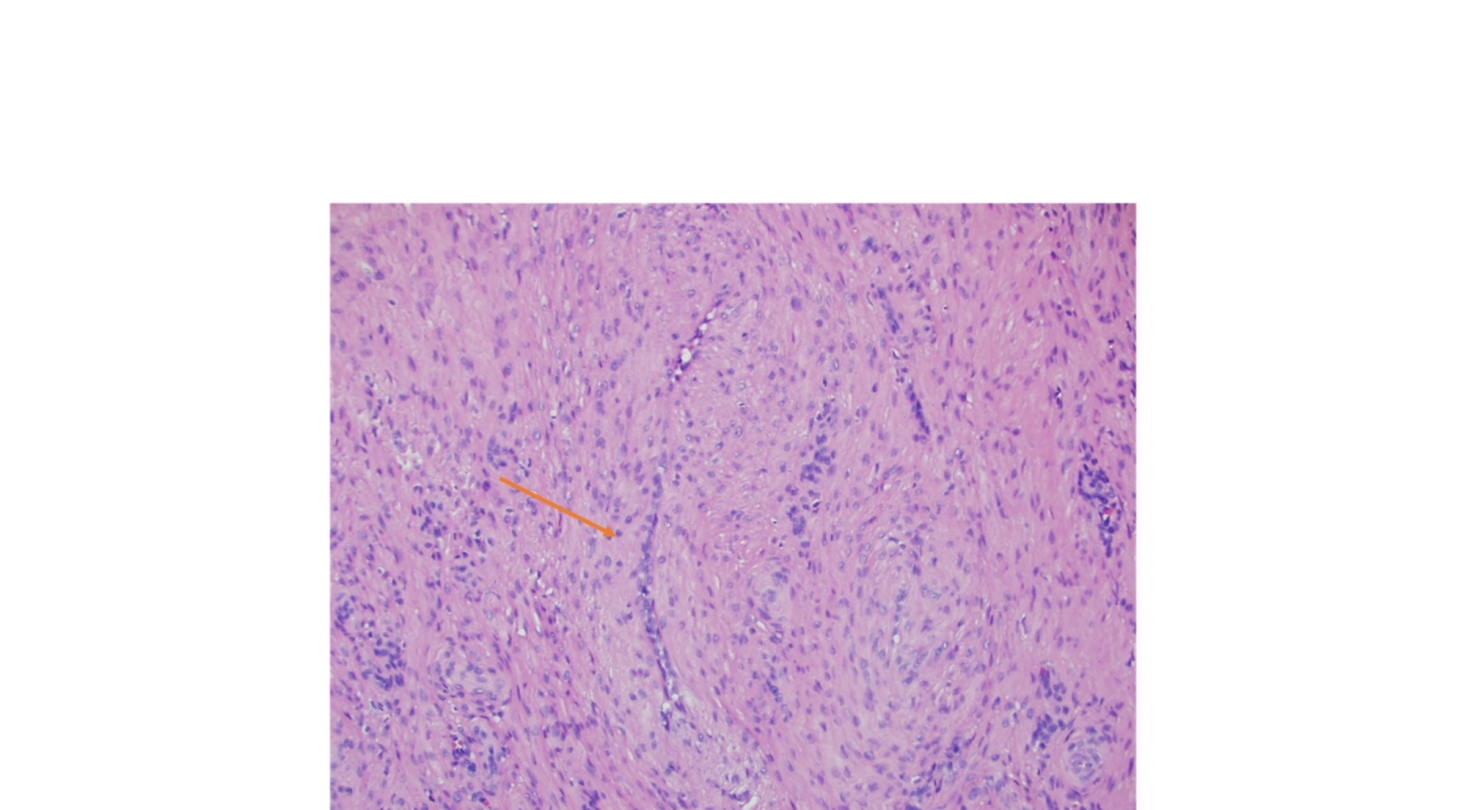
Microscopic exam revealed closely packed fascicles of spindle cell with myoid features surrounding compressed vascular lumina (20x)

A 68-year-old male presented with a two-year history of a cystic nodule on the radial aspect of the P1 (proximal) phalanx of the left third finger. The cyst had been progressively increasing in size over the last two years and became painful a few months prior to the presentation. He had no other associated symptoms. His past medical history was significant for HIV (well controlled, last CD4 700-900), chronic atrial fibrillation on dabigatran, diabetes mellitus type 2, essential hypertension and hyperlipidemia. Physical exam showed a 12 mm x 11 mm, nontender, mobile subcutaneous cystic lesion at the dorsal aspect of the left distal phalanx of the third finger. Complete blood counts, renal function, basic electrolytes and liver function were unremarkable. Given the location and clinical characteristics of the lesion, ganglion cyst, arteriovenous malformation, hemangioma, lipoma and fibroma were initially considered in the differential diagnosis. The patient opted for excision of the nodule. On macroscopic examination, the tumor was a well-circumscribed tan-pink nodular mass measuring 0.7 x 0.7 x 0.5 cm. Microscopic examination showed a solid type tumor with well-circumscribed closely packed smooth muscle fascicles with moderately low cellularity surrounding vascular lumina (Figures 1, 2).

**Figure 3:**
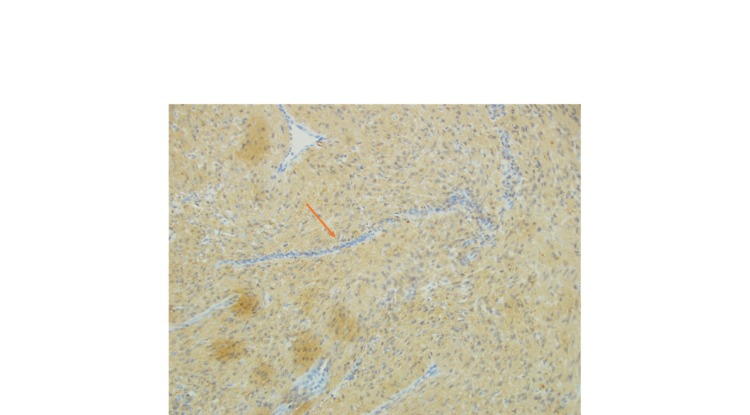
Specimen was stained positive with smooth muscle actin (SMA) depicting smooth muscle differentiation

**Figure 4:**
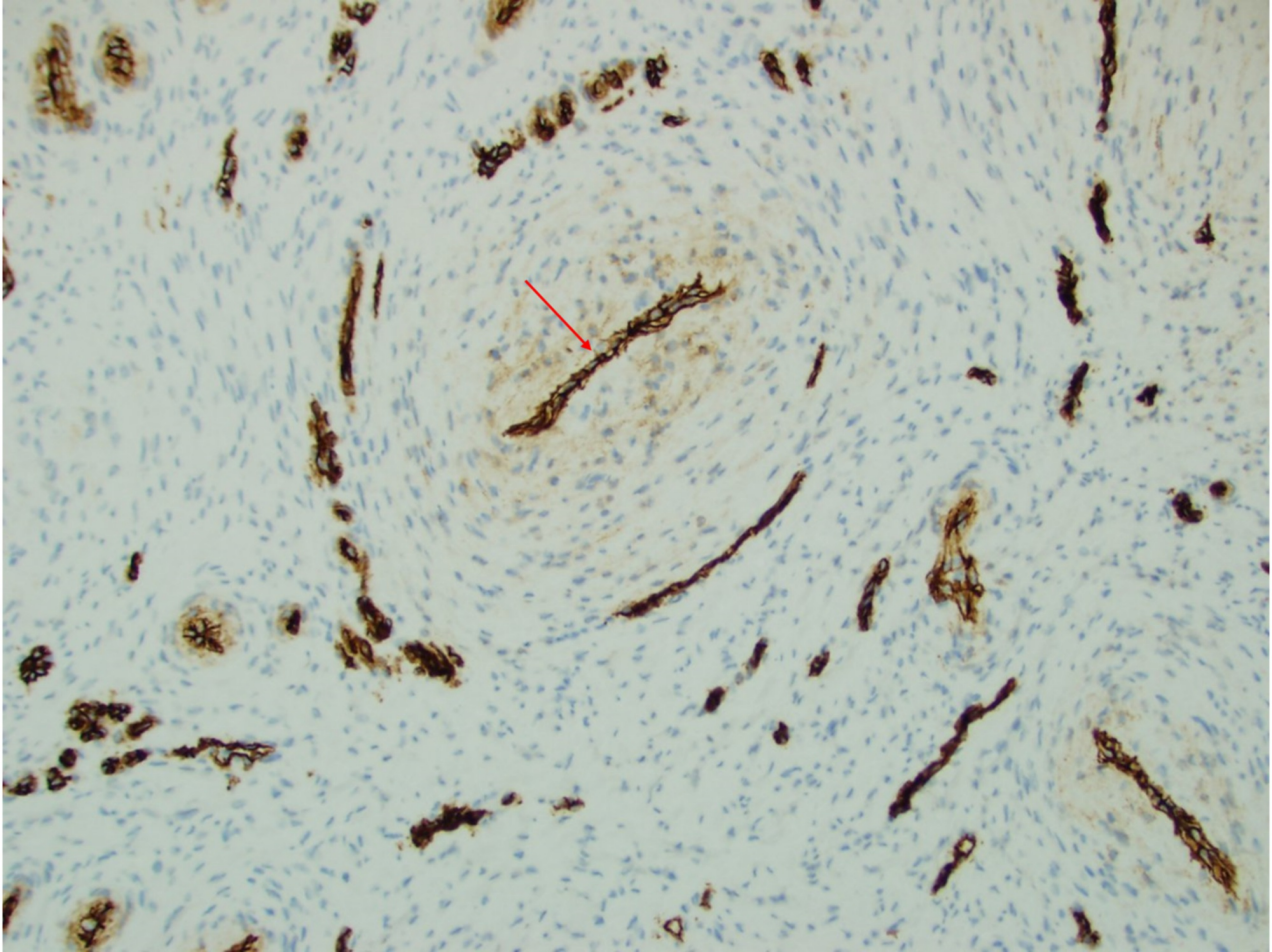
CD34 outlined the compressed endothelial cells surrounded by spindle cell proliferation in a concentric pattern

There was no evidence of atypia, hemorrhage, necrosis, mitosis or calcifications. Immunohistochemical staining with CD34 and smooth muscle actin were positive (Figures 3, 4). Testing for S100 (a neural tissue marker) was negative and desmin (a myogenic marker) was noncontributory. Diagnosis of angioleiomyoma was made after the histopathological exam. The patient recovered well from the procedure, and there was no recurrence after nine months of follow-up. 

## Discussion

Angioleiomyoma is a rare tumor consisting approximately 4.4% of all benign soft tissue masses [1]. It originates from the smooth muscle cells in blood vessels and involves mostly lower extremities. Hand involvement by angioleiomyoma is extremely rare due to paucity of smooth muscles in vasculature of the hand [2]. Epidemiologically, females are affected more than males with a ratio of 1.7:1, but upper extremity involvement was reported to be more common in males, as in the case of our patient [3,4]. Multiple theories regarding the etiology of angioleiomyoma have been proposed in the literature, including minor trauma, hamartomatous changes, venous stasis and hormonal imbalance [1,3]. 

Pain is the most common presenting complaint, occurring in 60% of cases [1,3,4]. The pain is thought to be a result of stretching of the nerves in the capsule of tumor, vasoconstriction giving rise to local ischemia or due to release of chemical mediators from mast cells [3,4]. They have an insidious growth over many years ranging from five to seven years in one study before the initial presentation [3]. 

Angioleiomyomas can be divided into three histologic subtypes: solid or capillary, venous and cavernous [3,5]. Solid type consists of closely compacted smooth muscle bundles that surround blood vessels; venous type contains vessels having thick muscular walls and are not so compact; and cavernous type incorporates dilated vascular channels with minimal smooth muscles [1-3]. These tumors have also been divided into two categories based on their location, namely tumors of the extremities and head region [3]. Tumors of the extremities are mainly of the solid type, whereas tumors of the head region are mainly of the venous type. The solid type is three times more common in females and the cavernous type is four times more common in males [3]. The tumor found in our case was of the solid type which is unusual in males. 

Angioleiomyomas are rarely diagnosed before excision and histopathological study conducted due to its rarity and vast differential diagnosis, including glomus tumor, traumatic neuroma, angiolipoma and hemangioma [1,6-8]. Due to rare cases of angioleiomyomas arising from blood vessels, preoperative imaging must be obtained to exclude that possibility to allow better surgical planning. The usual modality of treatment is surgical excision of the mass. Most cases of angioleiomyoma in the literature did not recur following excision, as is the case of our patient. Only two previously reported cases have recurred in the same location following surgery [3].

## Conclusions

Angioleiomyoma is an uncommon tumor. Due to rarity of the lesion and extensive differential diagnosis, it is commonly misdiagnosed before histopathological study. Therefore, it should be considered in the differential diagnosis in patients presenting with a painless subcutaneous nodule of the upper extremities.
